# Bifurcation Diagrams of Nonlinear Oscillatory Dynamical Systems: A Brief Review in 1D, 2D and 3D

**DOI:** 10.3390/e26090770

**Published:** 2024-09-09

**Authors:** Wieslaw Marszalek, Maciej Walczak

**Affiliations:** Department of Computer Science, Opole University of Technology, 45-758 Opole, Poland

**Keywords:** oscillatory signals, 0–1 test for chaos, sample entropy, largest Lyapunov exponent approach, bifurcation diagrams with one (1D), two (2D) and three (3D) varying parameters, electric arc system, calcium oscillatory system

## Abstract

We discuss 1D, 2D and 3D bifurcation diagrams of two nonlinear dynamical systems: an electric arc system having both chaotic and periodic steady-state responses and a cytosolic calcium system with both periodic/chaotic and constant steady-state outputs. The diagrams are mostly obtained by using the 0–1 test for chaos, but other types of diagrams are also mentioned; for example, typical 1D diagrams with local maxiumum values of oscillatory responses (periodic and chaotic), the entropy method and the largest Lyapunov exponent approach. Important features and properties of each of the three classes of diagrams with one, two and three varying parameters in the 1D, 2D and 3D cases, respectively, are presented and illustrated via certain diagrams of the *K* values, −1≤K≤1, from the 0–1 test and the sample entropy values SaEn>0. The *K* values close to 0 indicate periodic and quasi-periodic responses, while those close to 1 are for chaotic ones. The sample entropy 3D diagrams for an electric arc system are also provided to illustrate the variety of possible bifurcation diagrams available. We also provide a comparative study of the diagrams obtained using different methods with the goal of obtaining diagrams that appear similar (or close to each other) for the same dynamical system. Three examples of such comparisons are provided, each in the 1D, 2D and 3D cases. Additionally, this paper serves as a brief review of the many possible types of diagrams one can employ to identify and classify time-series obtained either as numerical solutions of models of nonlinear dynamical systems or recorded in a laboratory environment when a mathematical model is unknown. In the concluding section, we present a brief overview of the advantages and disadvantages of using the 1D, 2D and 3D diagrams. Several illustrative examples are included.

## 1. Introduction

Bifurcation diagrams of nonlinear dynamical systems can be of different natures, and various tools can be applied to create such diagrams. Typical diagrams showing oscillatory responses may use the largest Lyapunov expenent (LLE) method [1], 0–1 test for chaos [2,3,4,5,6] (see Appendix A) various definitions of entropy [7,8,9,10,11,12], statistical quantities and hypothesis testing [13,14,15,16,17] or machine learning methods [18]. These methods, in spite of the fact that they are based on different mathematical concepts, can be used to identify and classify the oscillatory behavior of nonlinear systems described by nonlinear ordinary differential equations (ODEs) or, sometimes, of the time series recorded in a laboratory setting when a mathematical model is unknown. The LLE method has been known for a long period of time and is well-researched. Entropy methods have been applied for the analysis and identification of time series, followed by the 0–1 test approach and hypothesis testing and machine learning approaches. Obvious questions arise when applying such different methods to a particular nonlinear system: do such methods provide the same (or similar) results and conclusions about the examined system? What internal parameters (or constants) should be chosen in these methods to derive and obtain an acceptable outcome about the system under investigation? For example, what parameters $n_{cut}$ and $\bar{N}$ (see Appendix A) should be chosen in the 0–1 test method to obtain the same conclusion when certain *m*, *r* and *N* values are used in the sample entropy (SaEn) method? Are the illustrative diagrams representing the behavior of nonlinear dynamical systems close to each other when different methods are used? This paper examines such issues, and, at least partially, attempts to provide a comparative analysis of the above-mentioned methods. This is one of the goals of the current analysis. Another goal is to look at those methods when we move from one varying parameter in a dynamical system to two, and further to three parameters, thus analyzing the same dynamical system in our notation of the 1D, 2D and 3D bifuraction diagrams.

Although the analyzed methods can be used in many nonlinear dynamical systems in engineering, science and economics, we decided to focus on two dynamical systems that differ significantly from each other. First, they come from different engineering and science areas (electrical and biochemical). Second, the mathematical models have different numbers of parameters that may vary. The electrical arc system (see Equation (A6) in Appendix B) has only three parameters, *R*, *L* and *C*, when *m* is kept constant, while the calcium oscillating system (see Appendix C) has seventeen parameters. The nonlinear systems under consideration are as follows:

- the electric arc RLC system described in ref. [18,19,20,21,22],
- the calcium system given in ref. [8,9,23,24,25,26].

**Figure 1:**
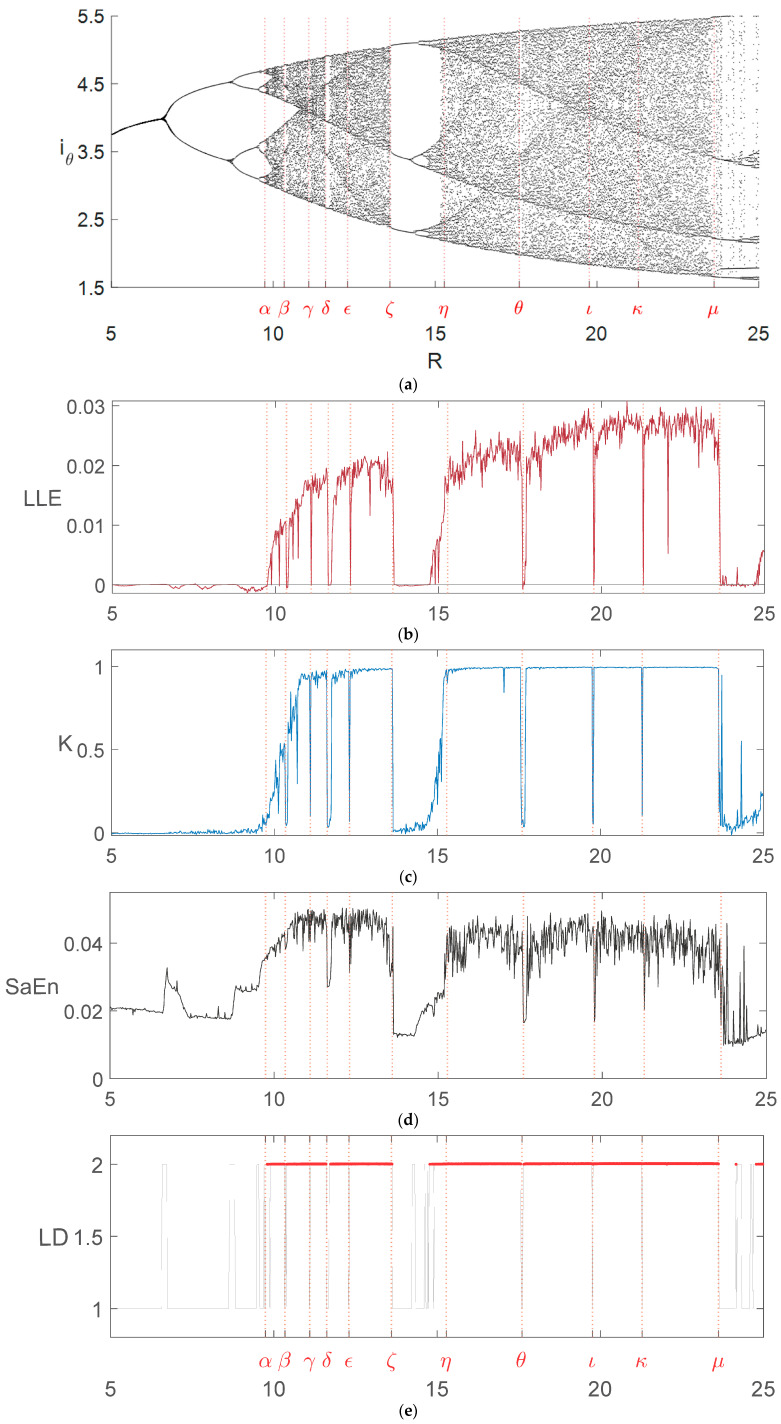
Five different 1D bifurcation diagrams for the *RLC* electric arc system [19] for the varying parameter 5≤R≤25. The integration fixed step-size in the Runge–Kutta IV method was dt=0.01. (**a**) Bifurcation diagram with local maximum values of periodic and chaotic responses for the nonlinear electric arc system. (**b**) Diagram of the LLE corresponding to the diagram in (**a**). (**c**) Diagram of the 0–1 test values *K* corresponding to the diagram in (**a**). Parameters given in Appendix A. (**d**) Sample entropy values SaEn corresponding to the diagram in (**a**). Parameters of the method were r=0.05, m=100 and N=10,000 (Appendix D). (**e**) Lyapunov dimension (LD) values corresponding to the diagram in (**a**). The LD values are greater than but close to 2 for the intervals marked with the red horizontal segments.

**Figure 2:**
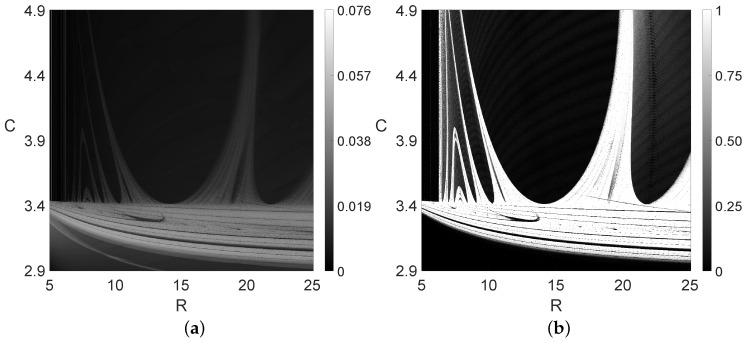
Two diagrams for the electric arc circuit with varying *R* and *C* parameters. (**a**) Sample entropy diagram with 0<SaEn<0.076. (**b**) The 0–1 test diagram with 0<K<1.

**Figure 3:**
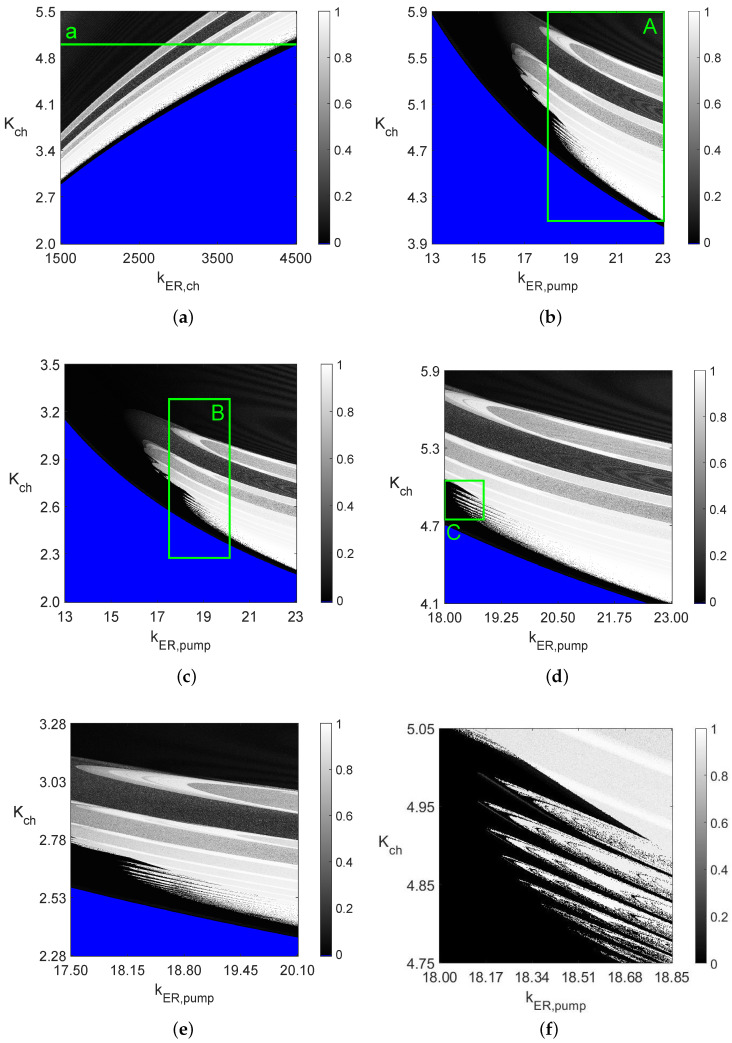
Two-parameter 1000×1000 diagrams from the 0–1 test, each obtained using the Runge–Kutta IV solver with dt=0.001 for 0≤t≤500 when the solution in the interval 300≤t≤500 and T=40 was used in the 0–1 test. Obtaining each of the above two-parameter diagrams requires solving the nonlinear system in ref. [8] $10^{6}$ times with additional computations (classification of the type of these solutions). A total of 256 gray levels were used for parameter *K* (along the vertical bars on the right-hand side of each diagram). (**a**) Varying $K_{ch}$ and $k_{ER,ch}$ (constant $k_{ER,pump}=20$). (**b**) Varying $K_{ch}$ and $k_{ER,pump}$ (constant $k_{ER,ch}=3500$). (**c**) Varying $K_{ch}$ and $k_{ER,pump}$ (constant $k_{ER,ch}=1000$). (**d**) Diagram in area *A* in (**b**). (**e**) Diagram in area *B* in (**c**). (**f**) Diagram in area *C* in (**d**).

**Figure 4:**
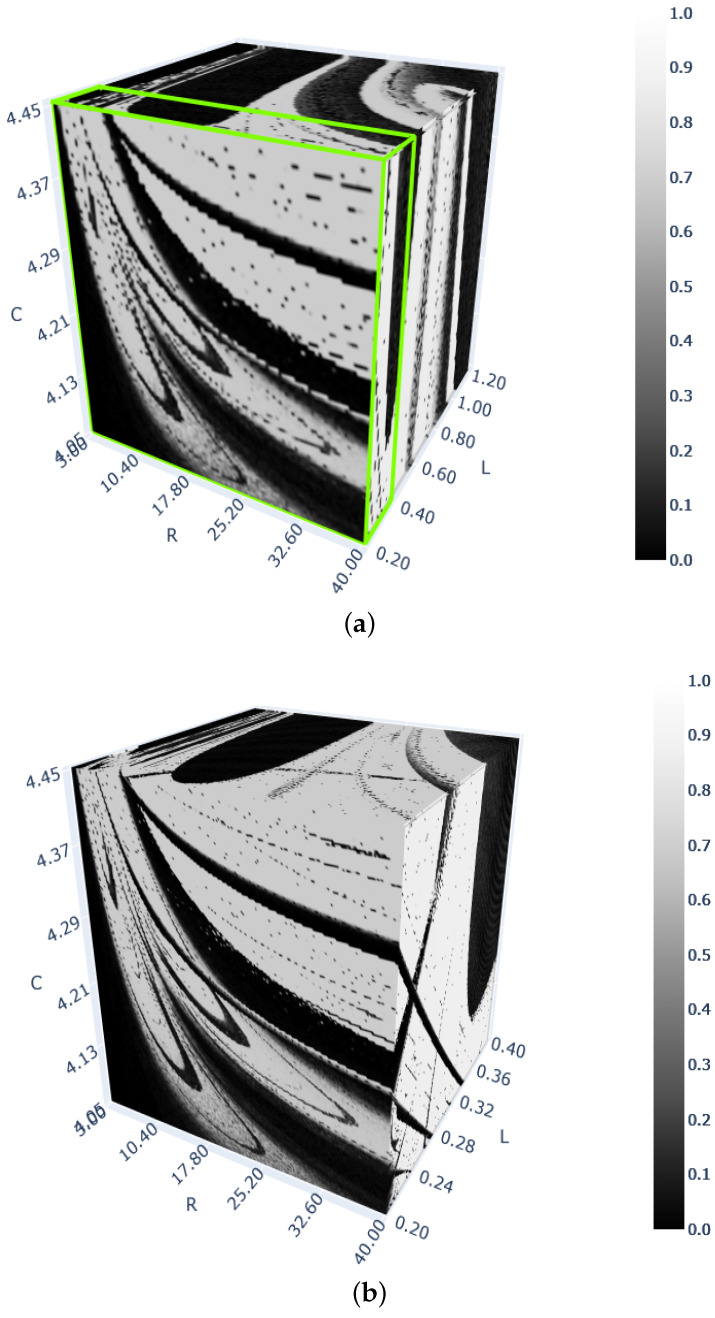
Three-dimensional diagrams of test 0–1 for chaos for the electric arc system. A total of 256 gray levels were used for parameter *K* (vertical bars). (**a**) Parameters R∈(3;40),C∈(4.05;4.45), L∈(0.2;1.2). Computations performed with $10^{6}$ discrete points in the box of size 100×100×100. (**b**) Parameters R∈(3;40), C∈(4.05;4.45),L∈(0.2;0.4). Computations performed with $8\times 10^{6}$ discrete points in the small green box in (**a**) (size 200×200×200).

**Figure 5:**
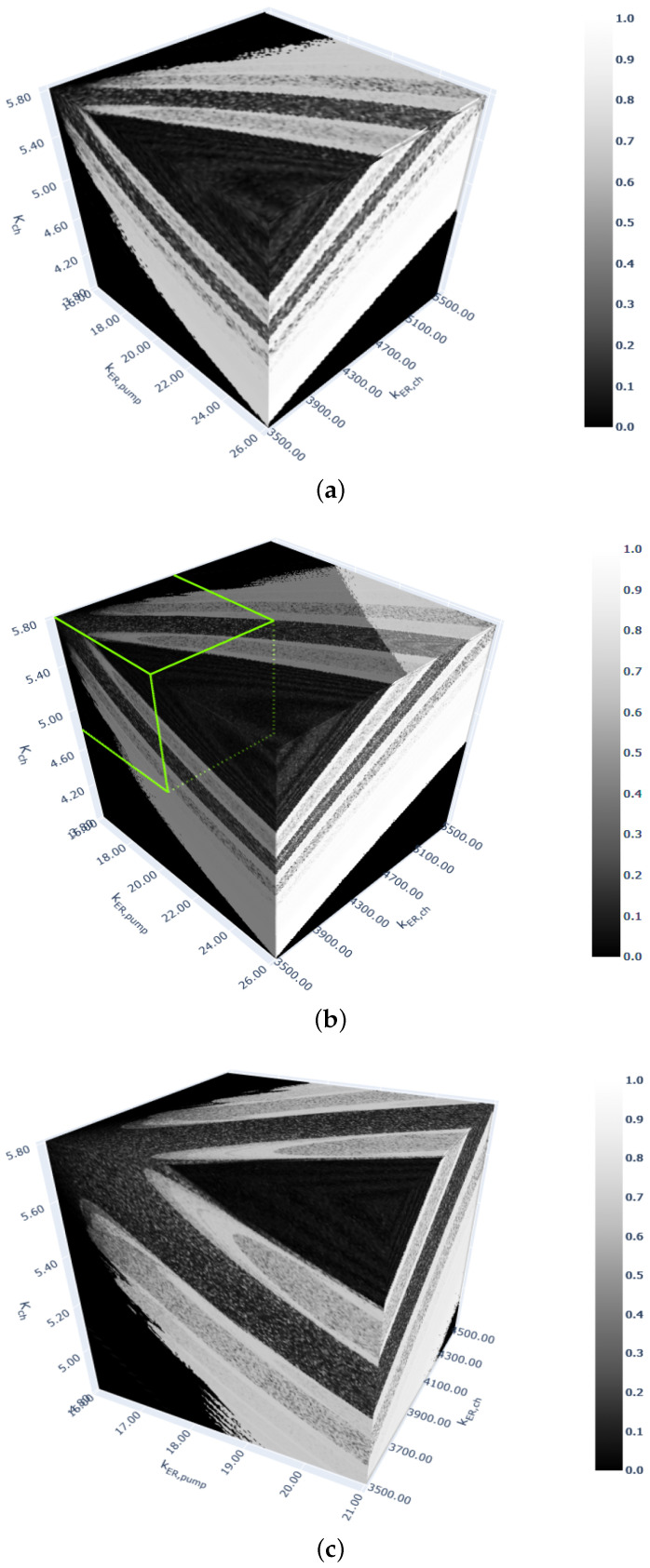
Three-dimensional bifurcation diagrams for the cytosolic calcium oscillation model. Computations performed with $10^{6}$ (**a**) and $8\times 10^{6}$ (**b**,**c**) discrete points. (**a**) $k_{ER,pump}\in \left(16;26\right),K_{ch}\in \left(3.8;5.8\right),k_{ER,ch}\in \left(3500;5500\right)$. (**b**) $k_{ER,pump}\in \left(16;26\right),K_{ch}\in \left(3.8;5.8\right),k_{ER,ch}\in \left(3500;5500\right)$. (**c**) $k_{ER,pump}\in \left(16;21\right),K_{ch}\in \left(3.8;4.8\right)$, $k_{ER,ch}\in \left(3500;4500\right)$ (the green box in (**b**)).

**Figure 6:**
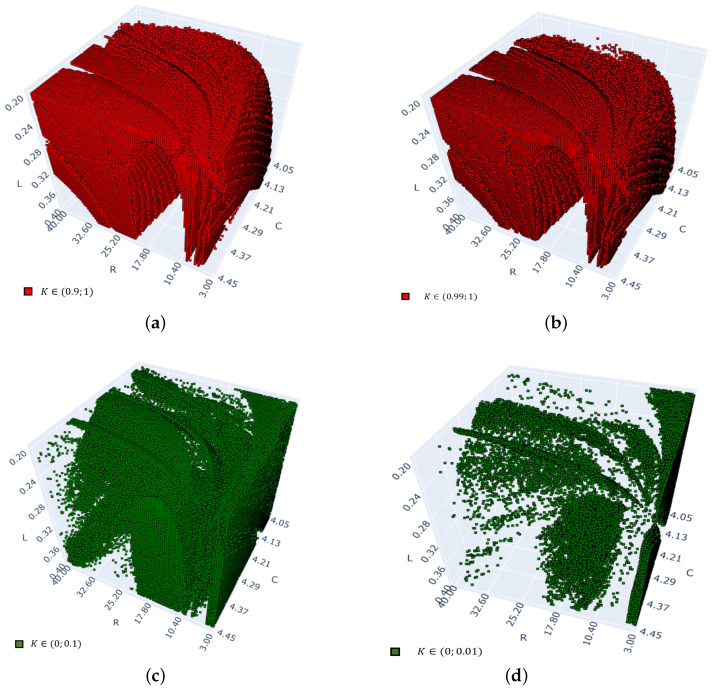
Three-dimensional diagrams of size 100×100×100 of the 0–1 test for the arc system with parameters R∈[3,40], C∈[4.05,4.45] and L∈[0.2,0.4]. Points representing chaotic responses with *K* values close to 1 are shown in (**a**,**b**). Points representing periodic responses with *K* values close to 0 are shown in (**c**,**d**). (**a**) Points in the cube with K∈(0.9;1.0). (**b**) Points in the cube with K∈(0.99;1.0). (**c**) Points in the cube with K∈(0;0.1). (**d**) Points in the cube with K∈(0;0.01).

**Figure 7:**
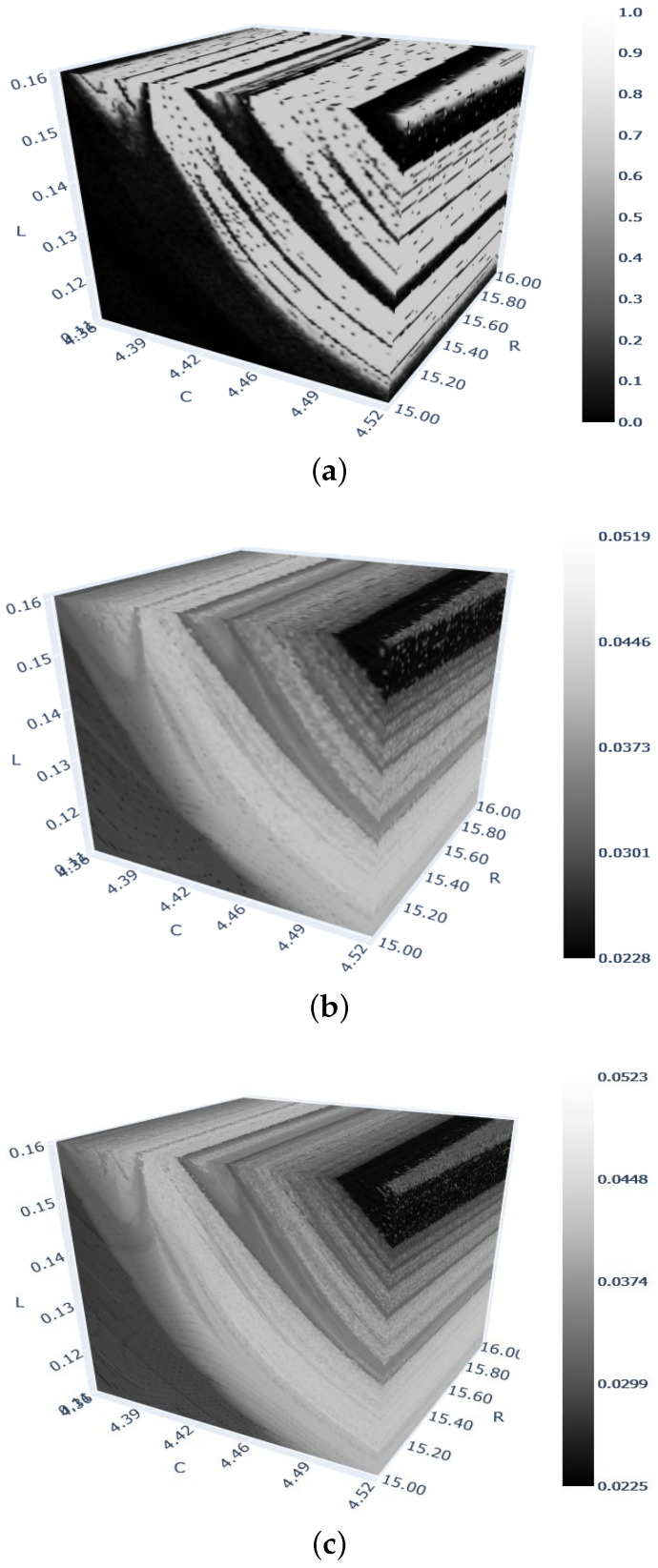
Three-dimensional diagrams of the 0–1 test and sample entropy methods for the electric arc system. A total of 256 gray levels were used for the values of sample entropy (vertical gray bars in (**b**,**c**). (**a**) Parameters R∈(15;16),C∈(4.36;4.52),L∈(0.11;0.16). Computations performed with $10^{6}$ points using the 0–1 test method. (**b**) Parameters as in (**a**). Computations performed with $10^{6}$ points using the sample entropy method. (**c**) Parameters as in (**a**). Computations performed with $8\times 10^{6}$ points using the sample entropy method.

We present five different diagrams in the 1D case in Figure 1, while applying the 0–1 test and sample entropy methods in these systems to the 2D and 3D cases in Figure 2, Figure 3, Figure 4, Figure 5, Figure 6 and Figure 7.

An exploration of diverse types of trajectories in nonlinear dynamical systems using the concepts of persistency, regularity, intermittency and transiency (in addition to chaos) is presented in a recent paper [27].

## 2. One-Dimensional Bifurcation Diagrams

Five typical 1D bifurcation diagrams are shown in Figure 1. These are different from the 1D diagrams for the nonlinear system presented in Appendix B, in which resistance *R* is treated as a variable parameter changing in the range R∈[5,25] with 1000 discrete values (step size ΔR=0.02). Two other parameters, the capacitance *C* and inductance *L*, were kept constant at C=3.14 and L=1. The vertical axis in Figure 1a shows the local maximum values of the current $i_{\theta }$ with the solutions (for each discrete value of *R*) obtained for t∈[0,2000], but with the period [0,500] being discarded. Thus, the identification of the maximum values was performed in the period t∈[500,2000]. The Runge–Kutta IV method was used to integrate the nonlinear system of ODEs describing the arc circuit. Figure 1b shows the corresponding LLE, while Figure 1c shows the diagram obtained using the 0–1 test with −1≤K≤1 and K≈0 indicating periodic and quasi-periodic responses, while K≈1 stands for chaotic ones. Next, Figure 1d depicts the sample entropy diagram. Finally, Figure 1e shows the LD diagram in which the red horizontal segments correspond to the chaotic intervals with the LD close to but greater than 2. Note that each of the above diagrams provides slightly different details about the obtained responses. For example, it is clear from Figure 1a that increasing *R* from the value of 5 to the middle of the interval [α,β] gives periodic responses with the period doubling bifurcation; that is, the period-1 response changes to period-2, then period-4, etc., eventually becoming a chaotic one. The period-5 response occurs around a narrow window with R=δ. Then, the period-3 responses occur for the values R>ζ, changing further to period-6, period-12, etc., with increased values of *R*. Analyzing the LLE diagram in Figure 1b, we obtain periodicity at the above-mentioned values of *R* without the period-doubling phenomenon. Moreover, based on Figure 1a, one can easily identify the maximum values of the periodic and chaotic responses, while such an identification based on the diagram in Figure 1b is not possible. For example, for R=7, we obtain the period-2 response with two local maximum values at around $i_{\theta ,max}$ equal 4.4 and 3.5. For *R* values slightly greater than ζ, we have a period-3 response with three local maximum values at around $i_{\theta ,max}$ equal to 5, 3.5 and 2.4. Overall, the diagram in Figure 1a seems to convey more information about the system under investigation than the diagram in Figure 1b.

The five types of 1D diagrams are not the only ones that can be drawn. Other possibilities are the bifurcation diagrams showing the changing frequency of oscillations, numbers (positive integers) of local maximum values in one period, or certain statistical parameters [6,14,15,16,17].

## 3. Moving from 1D to 2D Bifurcation Diagrams

Going from 1D to 2D diagrams with two changing parameters is often challenging computationally. Such calculations often require dedicated accelerators working in hierarchical environments and a hybrid programming model; e.g., the MPI+OpenMP [6]. When one parameter changes (as in Figure 1) with 1000 discrete values (note that the step size ΔR=0.02 in Figure 1) with two changing parameters, we obtain $10^{6}$ discrete points in some rectangular area of the changing parameters. For each discrete point (out of one million of them), we have to solve our nonlinear system, perform identification of the types of steady-state solutions and draw a color diagram representing these solutions. In this environment, the use of the diagram of the type shown in Figure 1a is problematic, as, in addition to the periodic/chaotic representation, we would have to add another dimension in which the local maxiumum values should be stored. This is impossible to do in the 2D case. Thus, one should rather use other types of diagrams; for example, the sample entropy method (see Appendix D) or the 0–1 test, as shown in Figure 2 and Figure 3. Figure 2 is interesting because it shows relatively similar (to the naked eye) diagrams obtained using conceptually different methods, namely the sample entropy and 0–1 test methods. Note that further, somewhat artificial adjustment (or rescaling) of the SaEn values could bring the sample entropy diagram very close to the 0–1 test diagram. The brighter areas in the sample entropy diagram would become close to the white areas in the 0–1 test diagram, while the darker areas in the sample entropy diagram would become close to the black areas in the 0–1 test diagram.

Figure 3 shows a series of 2D bifurcation diagrams of the nonlinear calcium model considered in ref. [8] (see Appendix C), which is quite interesting with a possibility of changing seventeen parameters. The two parameters used to obtain the diagrams in Figure 3 were the pairs of $K_{ch}$, $K_{ER,ch}$ and $k_{ER,pump}$ parameters, with various ranges of changes. Thus, the rectangular areas have different sizes, but each of the six diagrams in Figure 3 contains the *K* values from the 0–1 test for $10^{6}$ points, where 0≤K≤1. These values are represented by various shades of the grey color, as given by the vertical bars on the right-hand sides of each diagram. Other details of the computations are given in the captions in Figure 3. The blue color is used in Figure 3 to represent non-oscillatory solutions (constant steady-state values)—the solutions converge to constant stable equilibria for the parameters in the blue areas.

Note that having obtained the 2D diagrams in Figure 3, one can easily create 1D diagrams; for example, along line *a* in Figure 3a with fixed $K_{ch}$ and varying $1500\leq K_{ER,ch}\leq 4500.$ The obtained 1D diagram along the line *a* will be of the type shown in Figure 1b. Thus, each 2D diagram in Figure 3 corresponds to 1000 1D diagrams of the type shown in Figure 1b, as 1000 discrete points were used for the variable $K_{ch}$ between its lower value 2.0 and the highest value of 5.5.

As mentioned before in the Introduction, one can also create 2D bifurcation diagrams using other measures,;for example, the frequency diagrams or 2D arrays (with positive integers) representing the numbers of local maximum values in one period [9]. Other types of 2D bifurcation diagrams are also possible; for example, as an extension of the concept of the pseudo-periodic surrogate series to 2D and the hypothesis testing diagrams presented in ref. [14].

## 4. Three-Dimensional Bifurcation Diagrams

Moving one step further to three varying parameters, one can obtain 3D bifurcation diagrams. For example, if we simultaneously vary the three parameters used to obtain the diagrams in Figure 3 in some hypothetical cube of $K_{ch}\times k_{ER,ch}\times k_{ER,pump}$ with 1000 discrete points for each of the three parameters, then the 3D cube will contain $10^{9}$ discrete points. For each of these billion points, one has to solve the underlying nonlinear ODE system and identify the type of response (periodic, chaotic or constant). Computational effort increases exponentially.

Two 3D bifurcation diagrams (for the 0–1 test) for the nonlinear *RLC* arc system are shown in Figure 4, and three 3D diagrams for the nonlinear calcium system are shown in Figure 5. These diagrams were created using either 100×100×100 or 200×200×200 discrete points (see the captions of both figures). The diagram in Figure 4b of size 200×200×200 is the one for the range of the three parameters *R*, *L* and *C* determined by the green cube in Figure 4a. The entire diagram in Figure 4a of size 100×100×100 is of a poorer resolution compared to that in Figure 4b, which is clearly noticeable by comparing the quality of the front wall of the two diagrams (for L=0.2). Making suitable cuts along chosen planes in both 3D diagrams in Figure 4, one can obtain 2D diagrams in the same way one obtains a 1D diagram (of any type shown in Figure 1) from a respective 2D diagram.

The 3D diagrams in Figure 5 are for the nonlinear calcium system. Figure 5a,b show the responses in the same rectangular cube, but with different resolutions, while Figure 5c is the diagram in the rectangular green cube marked in Figure 5b. Note that the eight-fold increase in the number of discrete points in the cube in Figure 5b in comparison to the number of discrete points in the cube in Figure 5a results in an approximately eight-fold increase in the computation time, but it also provides better-quality results.

Next, the four diagrams in Figure 6 show only selected results from those shown in Figure 4a. Namely, Figure 6a includes only those points for which strongly chaotic responses were obtained with K∈[0.9,1], while Figure 6c includes only those points for which periodic responses with K∈[0,0.1] were detected. Similarly, Figure 6b includes only those points for which strongly chaotic responses were obtained with K∈[0.99,1], while Figure 6d includes only those points for which periodic responses with K∈[0,0.1] were detected. It is easy to notice that the points in both Figure 6a,c, when combined together, fill up almost the entire 3D cube. Thus, the test 0–1 indicates that other responses, those with K∈[0.1,0.9], are almost non-existent in the chosen 3D cube.

Finally, Figure 7 shows 3D bifurcation diagrams for the 0–1 test and sample entropy methods applied to the electric arc circuit. The two resolutions used clearly indicate that the eight-fold increase in the number of discrete points in the assumed cube leads to a better-quality diagram with smooth transitions between the sample entropy values. However, as shown in Appendix E, the computation time to obtain the diagram in Figure 7c is about 7.3 times greater than that in Figure 7b. On the other hand, with the same number of discrete points in the diagrams in Figure 7a,b, the sample entropy method requires about twice as much time as the 0–1 test method. Going one step further to see *the inside* of any 3D diagram (with millions of discrete points), one can design a relatively simple code to obtain several—say, 5, 10 or 20—2D crosscuts inside a cube (that is, 5, 10 or 20 two-parameter diagrams), rotate them at various angles to align them into preferred perspectives, and further zoom in on parts of the cube (3D diagram).

## 5. Conclusions

Bifurcation diagrams show different features of analyzed dynamical systems depending on the number of parameters being varied—one, two or three—as well as of the type of diagram, as illustrated in Figure 1 and Figure 2. Is it also possible to create other types of diagrams; for example, using the pseudo-periodic surrogates and hypothesis testing parameters from statistics or machine learning-based methods [13,14,18]. In certain cases, using two different types of diagrams, one can obtain almost identical results; for example, considering the periodicity–chaoticity, as shown in Figure 1. In other cases, it is difficult to compare the diagrams when using parameters of different natures and mathematical foundations; for example, the 0–1 test and sample entropy. One can find an interesting comparison of the diagrams obtained using the 0–1 test and sample entropy in ref. [7,9]. The difficulty in comparing is due to the uncertainty of the correctness of the chosen parameters in both methods; for example, the choice of the *m*, *r* and *N* parameters in the sample entropy method [7].

Another important issue is the computational effort needed to create the diagram. The effort (time of computation, storage requirement) increases exponentially with moving from 1D to 2D and then further to 3D. With increased resolutions (decreased step size of the changing parameters), in 2D and 3D, one should consider parallelization of computation to decrease the computational effort; preliminary results have been reported in [20,21]. The computational times for the 3D diagrams presented in this paper are shown in Appendix E. When choosing a particular method for analyzing and identifying time series, one needs to consider the computational time and memory requirements, the availability of computational codes and types of machines, as well as what information should be derived regarding the analyzed time series. Notice, for example, that the parallel computation system needed approximately 477,600 s (=132.7 h = 5.5 days) to create just one 200×200×200 diagram, shown in Figure 7c.

## Figures and Tables

**Figure A1:**
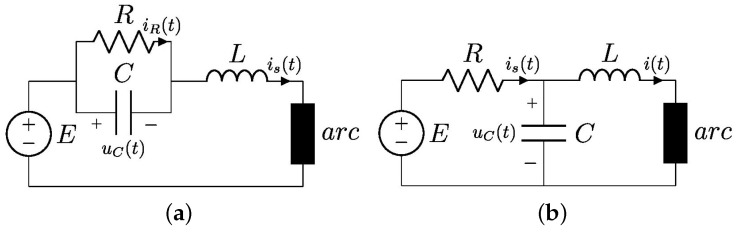
Electric arc circuits. Circuit B is also described by Equation (A6) with a suitable change in variables. (**a**) Circuit A. (**b**) Circuit B.

## Data Availability

Interested readers are welcome to request the individual codes used to create the bifurcation diagrams presented in this paper from the authors.

## Appendix A. The 0–1 Test (For Chaos)

The 0–1 test was developed by Gottwald and Melbourne [2,3,4]. Certain problems using the 0–1 test for continuous dynamical systems have been reported in refs. [5,6]. Here, a concise summary of the test is provided.

The test results have two forms: a single real number, *K*, and a two-dimensional graph of variables $\left(p_{c}\left(n\right),q_{c}\left(n\right)\right)$ [4,5]. When a chaotic sequence is fed into the test, *K* should be close to 1, whereas for periodic and quasi-periodic sequences, *K* should be close to 0. There are two methods of computing *K*: regression or correlation. For a time series $\left\{N_{k}\right\}$, $k=0,\ldots ,\bar{N}-1$ with the recommended value $\bar{N}=5000$, the $p_{c}$ and $q_{c}$ values are computed as follows:

(A1) $$p_{c}\left(n\right)=\sum_{j=0}^{n}N_{j}cos\left[\left(j+1\right)c\right],\;q_{c}\left(n\right)\;=\sum_{j=0}^{n}N_{j}sin\left[\left(j+1\right)c\right]$$

with $n=0,\ldots ,\bar{N}-1$ and a randomly chosen real number c∈(0,π). Then, the quantity $M_{c}\left(n\right)$, $n=0,1,\ldots ,n_{cut}$, called the mean square displacement of $p_{c}\left(n\right)$ and $q_{c}\left(n\right)$, is computed as follows:

(A2) $$M_{c}\left(n\right)=\lim_{\bar{N}\to \infty }\frac{1}{\bar{N}-1}\sum_{j=0}^{\bar{N}-1}{\left[p_{c}\left(j+n\right)-p_{c}\left(j\right)\right]}^{2}+{\left[q_{c}\left(j+n\right)-q_{c}\left(j\right)\right]}^{2}\;$$

with the recommended value $n_{cut}\approx \left(\bar{N}-1\right)/10$. If the regression method is applied, then the $K_{c}$ value, the asymptotic growth rate of the mean square displacement, is computed as follows:

(A3) $$K_{c}=\lim_{n\to \infty }\frac{log\;M_{c}\left(n\right)}{log\;n}.$$

For the correlation method, we create two vectors:

(A4) $$\begin{array}{ccc} \xi & = & \left(0,1,2,\ldots ,n_{cut}\right)\\ \Delta & = & (M_{c}\left(0\right),M_{c}\left(1\right),M_{c}\left(2\right),\ldots ,M_{c}\left(n_{cut}\right)), \end{array}$$

and the correlation coefficient $K_{c}$ is obtained as follows:

(A5) $$K_{c}=corr\left(\xi ,\Delta \right)\equiv \frac{cov(\xi ,\Delta )}{\sqrt{var(\xi )var(\Delta )}}$$

with *cov* and *var* denoting their covariance and variance, respectively [2,3]. In both the regression and correlation methods, the above steps are repeated for $N_{c}$ values of *c* chosen randomly from the interval (0,π). It is recommended that $N_{c}=100$. Computing the median of the $N_{c}$ values of $K_{c}$ yields the number *K*. All sequences tested in this paper using the 0–1 test had a length of 5000 real values (recommended in [2,4]). The special parameter *T* used to avoid the oversampling problem in the 0–1 test [28] was 40 for the diagrams shown in Figure 3 and 1700 for all other 0–1 test diagrams presented in this paper. We used the correlation method in all our computations involving the 0–1 test in this paper.

## Appendix B. The Electric Arc System

The oscillating electric arc RLC circuits shown in Figure A1 are described by a system of three ODEs on the left side of Equation (A6) below, and its dimensionless version is on the right side [18,19,20,21]:

(A6) $$\begin{array}{ccc} \begin{array}{ccc} \frac{di}{d\tau } & = & \frac{1}{L}\left(u_{C}-\frac{U(i_{\theta })}{i_{\theta }}i\right)\\ \frac{du_{C}}{d\tau } & = & \frac{1}{RC}\left(E-u_{C}-Ri\right)\\ \frac{di_{\theta }^{2}}{d\tau } & = & \frac{1}{\theta }\left(i^{2}-i_{\theta }^{2}\right) \end{array} & \to & \begin{array}{ccc} \frac{dx}{dt} & = & \frac{1}{L}\left(y-xz^{m}\right)\\ \frac{dy}{dt} & = & \frac{1}{RC}\left(R+1-y-Rx\right)\\ \frac{dz}{dt} & = & x^{2}-z \end{array} \end{array}$$

where $x=i/I_{0}$, $y=u_{C}/U_{0}$, $z=i_{\theta }^{2}/I_{0}^{2}$ and $i_{\theta }$, *i*, $u_{C}$ are the arc current, current through *L* and voltage across *C*, respectively. $i_{s}$ stands for the *source* current in Figure A1, while $U_{0}$ and $I_{0}$ are two constants from the *static* arc voltage–current characteristic $U\left(i_{\theta }\right)=U_{0}{\left(i_{\theta }/I_{0}\right)}^{m}$ with m<0.

To solve the dimensionless version of Equation (A6), we used the Runge–Kutta IV method with the time horizon 0≤t≤2000, integration fixed step dt=0.01 and initial conditions [x(0),y(0),z(0)]=[0.5,4.0,1.0] and m=−1/3. The ranges of parameters *R*, *L* and *C* are given in the captions of the various figures in this paper.

## Appendix C. Model of Cytosolic Calcium Oscillations

One of the store models considered in the literature for complex Ca^2+^ oscillations, with mitochondria included, is the model considered in refs. [23,24,25,26]. The three compartments considered are the cytosol, endoplasmic reticulum (ER) and mitochondria, yielding the three dynamical variables of free Ca^2+^ concentration $Ca_{cyt}$, $Ca_{ER}$ and $Ca_{m}$, respectively (see Figure 1 in [24]). Denoting $x_{1}=Ca_{cyt}$, $x_{2}=Ca_{ER}$ and $x_{3}=Ca_{m}$, the nonlinear autonomous system is as follows:

(A7) $$\begin{array}{ccc} x_{1}^{\prime } & = & J_{ER,ch}-J_{ER,pump}+J_{ER,leak}+J_{CaPr}-J_{Pr}\\ \; & \; & +\frac{\rho_{m}}{\beta_{m}}\left(J_{m,out}-J_{m,in}\right)\\ x_{2}^{\prime } & = & \frac{\beta_{ER}}{\rho_{ER}}\left(J_{ER,pump}-J_{ER,leak}-J_{ER,ch}\right)\\ x_{3}^{\prime } & = & J_{m,in}-J_{m,out} \end{array}$$

where ′ stands for the time derivative, and the following apply:

(A8) $$\begin{array}{ccc} J_{ER,ch} & = & k_{ER,ch}\frac{x_{1}^{2}}{K_{ch}^{2}+x_{1}^{2}}\left(x_{2}-x_{1}\right)\\ J_{ER,pump} & = & k_{ER,pump}x_{1}\\ J_{ER,leak} & = & k_{ER,leak}\left(x_{2}-x_{1}\right)\\ J_{CaPr} & = & k_{-}CaPr\\ J_{Pr} & = & k_{+}x_{1}Pr\\ Ca_{tot} & = & x_{1}+\frac{\rho_{ER}}{\beta_{ER}}x_{2}+\frac{\rho_{m}}{\beta_{m}}x_{3}+CaPr\\ Pr_{tot} & = & Pr+CaPr\\ J_{m,in} & = & k_{m,in}\frac{x_{1}^{n}}{K_{m}^{n}+x_{1}^{n}}\\ J_{m,out} & = & k_{m,out}x_{3} \end{array}$$

with the parameters *n*, $k_{ER,ch}$, $K_{ch}$, $k_{ER,pump}$, $k_{ER,leak}$, $k_{-}$, $k_{+}$, $Ca_{tot}$, $\rho_{ER}$, $\beta_{ER}$, CaPr, $Pr_{tot}$, Pr, $\rho_{m}$, $\beta_{m}$, $k_{m,in}$, $k_{m,out}$ and $K_{m}$ all being positive. These parameters denote various physical quantities in the above model [24].

After substituting Equation (A8) into Equation (A7), we obtain the following:

(A9) $$\begin{array}{ccc} x_{1}^{\prime } & = & k_{ER,ch}\frac{x_{1}^{2}}{K_{ch}^{2}+x_{1}^{2}}\left(x_{2}-x_{1}\right)-k_{ER,pump}x_{1}+\\ \; & \; & k_{ER,leak}\left(x_{2}\;-\;x_{1}\right)+\\ \; & \; & k_{-}\left(Ca_{tot}\;-\;\;x_{1}\;\;-\;\frac{\rho_{ER}}{\beta_{ER}}x_{2}\;-\;\frac{\rho_{m}}{\beta_{m}}x_{3}\right)-\\ \; & \; & k_{+}\left(Pr_{tot}-Ca_{tot}+x_{1}+\frac{\rho_{ER}}{\beta_{ER}}x_{2}+\frac{\rho_{m}}{\beta_{m}}x_{3}\right)x_{1}+\\ \; & \; & \frac{\rho_{m}}{\beta_{m}}\left(k_{m,out}x_{3}-k_{m,in}\frac{x_{1}^{n}}{K_{m}^{n}+x_{1}^{n}}\right)\equiv {\tilde{f}}_{1}\left(x_{1},x_{2},x_{3},p\right)\\ x_{2}^{\prime } & = & \frac{\beta_{ER}}{\rho_{ER}}(k_{ER,pump}x_{1}-k_{ER,leak}\left(x_{2}-x_{1}\right)-\\ \; & \; & k_{ER,ch}\frac{x_{1}^{2}}{K_{ch}^{2}+x_{1}^{2}}\left(x_{2}-x_{1}\right))\equiv {\tilde{f}}_{2}\left(x_{1},x_{2},p\right)\\ x_{3}^{\prime } & = & k_{m,in}\frac{x_{1}^{n}}{K_{m}^{n}+x_{1}^{n}}-k_{m,out}x_{3}\equiv {\tilde{f}}_{3}\left(x_{1},x_{3},p\right) \end{array}$$

Notice the presence of many parameters denoted by vector *p* in Equation (A9) and the nonlinear terms of polynomial and rational types. All computations presented in this paper were performed with the following parameters kept constant: n=8, $k_{ER,leak}=0.05\;s^{-1}$, $\rho_{ER}=0.01$, $\beta_{ER}=0.0025$, $k_{-}=0.01\;s^{-1}$, $k_{+}=0.1\;\mu M^{-1}s^{-1}$, $Ca_{tot}=90\;\mu M$, $Pr_{tot}=120\;\mu M$, $\rho_{m}=0.01$, $\beta_{m}=0.0025$, $k_{m,in}=75\;\mu M\;s^{-1}$, $k_{m,out}=0.1265625\;s^{-1}$, $K_{m}=0.8\;\mu M$. The varying parameters are identified in the bifurcation diagrams. The zero initial conditions for the three variables $x_{i}$, i=1,2,3 were used. The Runge–Kutta IV *ode45* solver was used with the fixed value dt=0.001 (the output step size), $abserr=relerr=10^{-8}$, 0≤t≤1000, and the analyzed discrete time-series values were taken from the second half of that period.

## Appendix D. Sample Entropy Concept [9]

The entropy concept, and the sample entropy in particular [7], is used mainly in physics and medicine (physiology, cardiology and enzymology), and to a lesser extent, in engineering. The primary use of entropy is to determine a disorder of time-series, mainly due to the random noise present within the analyzed time series. Signals with low sample entropy values have more regularity, while those with higher entropy values are termed disordered, or signals of a higher complexity. Similarly to the 0–1 test, the difficulties in computing sample entropy values for time series have been reported [10,11,12]. The three parameters used in the calculation of the sample entropy are *m*, the length of a short epoch of data or template of points; *r*, the tolerance of identifying “similar” epochs (usually r∈[0.05,0.20]; that is, 5–20% of the signal’s standard deviation); and *N*, the length of the time series being analyzed. The basic formula for sample entropy SaEn with the three parameters *m*, *r* and *N* is as follows:

(A10) $$SaEn=-log(\sum A_{i}/\sum B_{i})$$

where $A_{i}$ is a number of matches of epochs of length m+1 for the *ith* template, and $B_{i}$ is a number of matches of length *m*.

We chose to apply the *SaEn* algorithm, since the sample entropy concept presented briefly above is more appealing, easy to explain and more effective in computation than other entropy concepts; for example, the approximate entropy *ApEn*.

## Appendix E. Computational Environment

All computations of the 3D diagrams in this paper were performed using Intel Phi MIC (Many Integrated Core) and parallel calculations. The system consisted of three cards of (PCI) Intel Xeon Phi Coprocessor 7120. Each of the cards had 61 cores clocked at 1.238 GHZ, and each core enabled four threads. This allowed the application of 244 threads on each card. The code on one core could be executed sequentially, as well as through a two-pipeline processing. Each card was equipped with 16 GB of RAM. Communication with other devices and cards was facilitated by the PCI bus. The Intel MIC architecture was installed on the main board Supermicro X10DRG-OT+-CPU with two Intel Xeon ES-2650 v.3 2.30GHZ processors and 112 GB of RAM. The operating system was 64 bit CentOS 7.6.1810, and the installed cards were available as three Linux hosts in the TCP/IP network.

Moreover, the computations were performed in C and C++, and the Intel MPI Library and OpenMP were used to parallelize the computations. The compilation was performed by the Intel(R) C++ Compiler (icc) with the MIC architecture (e.g., vectorization).

In the above described environment, the computation times for the bifurcation diagrams were as follows:

- Figure 4a, the 0–1 test with 100×100×100 points: 25,007 s.
- Figure 4b, the 0–1 test with 200×200×200 points: 200,891 s.
- Figure 5a, the 0–1 test with 100×100×100 points: 4717 s.
- Figure 5b, the 0–1 test with 200×200×200 points: 36,989 s.
- Figure 5c, the 0–1 test with 200×200×200 points: 37,303 s.
- Figure 7a, the 0–1 test diagram with 100×100×100 points: 28,275 s.
- Figure 7b, the sample entropy method with 100×100×100 points: 65,212 s.
- Figure 7c, the sample entropy method with 200×200×200 points: 477,603 s.

Note that increasing the number of discrete points eight-fold resulted in approximately the same increase in the computational times while improving the quality of the obtained diagrams. It is a matter of subjective observation whether or not the eight-fold increase in computational times is worth the obtained quality of the 3D diagrams.
